# Evaluating Scopus AI Versus Traditional Searches for Literature Review About Prepectoral Breast Reconstruction: An Exploratory Study

**DOI:** 10.3390/medsci13040211

**Published:** 2025-10-01

**Authors:** Ionuț Ștefan Ciule, Ariana-Anamaria Cordos, Răzvan Alexandru Ciocan, Andra Ciocan, Maximilian Vlad Muntean, Claudia Diana Gherman

**Affiliations:** 1 Department of Plastic and Reconstructive Surgery, “Prof. Dr. I. Chiricută” Institute of Oncology, 400015 Cluj-Napoca, Romania; 2 Department of Plastic and Reconstructive Surgery, “Iuliu Hațieganu” University of Medicine and Pharmacy, 400012 Cluj-Napoca, Romania; 3 Department of Public Health, Faculty of Political, Administrative and Communication Sciences, Babeș-Bolyai University, 400084 Cluj-Napoca, Romania; 4 Romanian Society of Medical Informatics, 300222 Timisoara, Romania; 5 Department of Surgery–Practical Abilities, “Iuliu Hațieganu” University of Medicine and Pharmacy, 400006 Cluj-Napoca, Romania; 6 Second Department of Surgery, “Iuliu Hațieganu” University of Medicine and Pharmacy, 400006 Cluj-Napoca, Romania; 7 “Prof. Dr. Octavian Fodor” Regional Institute of Gastroenterology and Hepatology Cluj-Napoca, Croitorilor Street, No. 19-21, 400162 Cluj-Napoca, Romania; andra.ciocan10@gmail.com

**Keywords:** breast reconstruction, mammaplasty, breast implantation, generative artificial intelligence, literature review

## Abstract

**Background**: Artificial intelligence tools are increasingly being used to assist literature reviews, but their effectiveness compared to traditional methods is not well established. This study compares Scopus AI with PubMed keyword searches on the topic of primary prepectoral breast reconstruction after radical mastectomy. **Methods**: On 28 May 2025, two literature searches were conducted on the topic of primary prepectoral breast reconstruction after radical mastectomy—one using Scopus AI and the other using manual keyword searches in PubMed. Both searches were limited to peer-reviewed clinical studies in English, excluding case reports and studies with fewer than 10 patients. Data extracted included study design, sample size, outcomes, and key findings. **Results**: The Scopus AI search retrieved 25 articles, while the traditional method identified 4. After removing duplicates, non-English texts, and non-relevant sources, 17 articles were included in the final analysis. Scopus AI provided automatic summaries, while manual review was required for the traditional method. No overlap was found between the two methods. **Conclusions**: AI tools like Scopus AI can enhance the speed and breadth of literature reviews, but human oversight remains essential to ensure relevance and quality. Combining AI with traditional methods may offer a more balanced and effective approach for clinical research.

## 1. Introduction

In recent years, the advent of AI-powered tools in academia have revolutionized the way researchers conduct literature reviews. These tools promise to deliver comprehensive reviews almost instantaneously, leveraging advanced algorithms to sift through vast amounts of data. However, as these AI tools become more prevalent, it is crucial to understand their methodologies, strengths, and limitations compared to traditional approaches. This study compares the outcomes of literature searches performed with Scopus AI versus traditional PubMed keyword searches, in order to assess their efficiency, comprehensiveness, and relevance. In this way, we seek to provide insights into the accuracy, relevance, and comprehensiveness of AI-driven reviews, ultimately guiding researchers in selecting the most appropriate tools for their work and ensuring the integrity of academic research.

We selected primary prepectoral breast reconstruction as the test topic because it represents an emerging surgical technique characterized by a growing but still relatively limited body of evidence. This setting provided an opportunity to assess whether AI-assisted tools are capable of efficiently identifying both well-established studies and more recent publications within a focused and evolving clinical domain.

## 2. Materials and Methods

### 2.1. Objective

The objective of this study is to compare the effectiveness and outcomes of literature reviews conducted using Elsevier. (2025). Scopus AI [Generative AI tool] and traditional PubMed keyword searches, specifically focusing on the topic of “Primary prepectoral breast reconstruction after radical mastectomy”.

### 2.2. Study Selection

Two authors independently evaluated titles and abstracts against predefined inclusion and exclusion criteria. Full-text assessment was conducted when relevance could not be determined from the abstract alone. Discrepancies were resolved through consensus.

### 2.3. Search Strategy

All searches were last updated on 28 May 2025, in order to capture the most recent publications available at the time of analysis. This update was performed to enhance the currency of the review and to ensure that the findings reflect the latest developments in the field. Restricting the search to a single cutoff date ensured methodological consistency across approaches. However, this also represents a limitation, as studies published thereafter may not have been captured.

#### 2.3.1. Scopus AI Methodology

Scopus AI was utilized to conduct the literature search. The AI’s natural language processing capabilities were employed to input the specific topic: “Primary prepectoral breast reconstruction after radical mastectomy”. The search was limited to articles published in peer-reviewed journals, focusing on clinical outcomes, patient safety, and the efficacy of prepectoral breast reconstruction. Non-English articles, case reports, and studies with fewer than 10 patients were excluded. Data extracted from the retrieved articles included study design, sample size, measured outcomes, and key findings.

#### 2.3.2. Traditional Keyword Search

**Figure 1:**
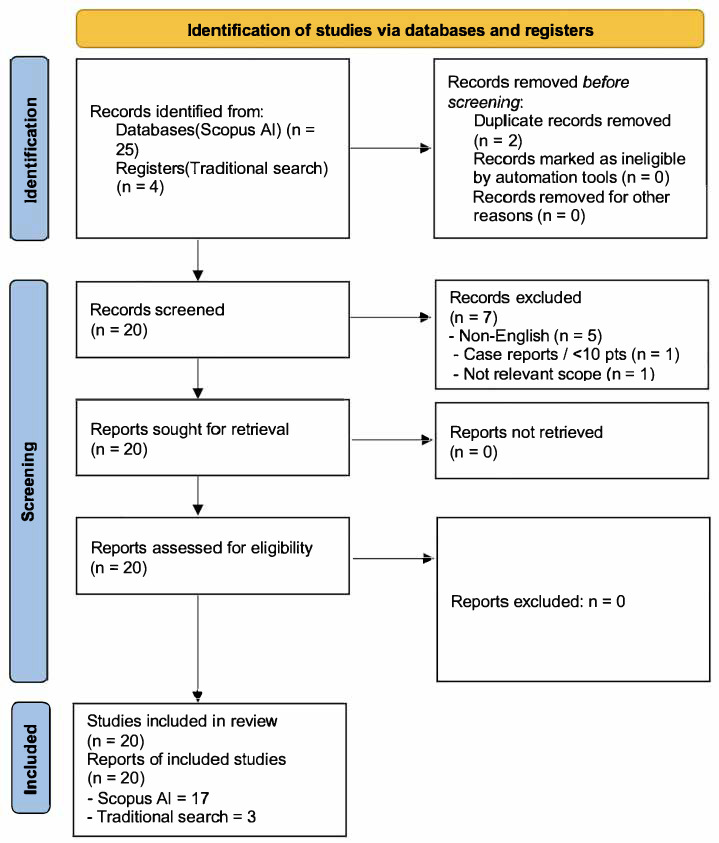
Summary of the findings.

A traditional keyword search was conducted using PubMed, Web of Science, and Wiley databases. The search terms employed were “prepectoral breast reconstruction” and “radical mastectomy.” The inclusion and exclusion criteria were identical to those used in the Scopus AI search. Similarly to the AI-based search, data on study design, sample size, measured outcomes, and key findings were extracted from the retrieved articles (Figure 1).

### 2.4. Comparison of Methodologies

#### 2.4.1. Efficiency

The efficiency of both methods was assessed by measuring the time taken to complete the literature search and comparing the number of relevant articles retrieved. The time taken to conduct the search and the number of relevant articles identified were recorded for both the Scopus AI and traditional keyword search methods.

#### 2.4.2. Quality of Results

The quality of the results obtained from both methods was evaluated based on two key factors: relevance and comprehensiveness. The relevance of the retrieved articles to the research topic was assessed. Additionally, the comprehensiveness of the literature review was evaluated by considering the coverage of key studies and findings.

#### 2.4.3. Analysis of Results

The data extracted from the articles retrieved using both methods were synthesized to identify common themes and patterns. The findings from the literature reviews conducted using Scopus AI and traditional keyword searches were compared to assess any discrepancies or similarities. Furthermore, the perspectives provided by the articles on the topic of primary prepectoral breast reconstruction, including patient outcomes, safety, and efficacy, were analyzed.

## 3. Results

The number of articles found using Scopus AI was 25, while the traditional keyword search yielded 4 articles (1 in Wiley, 1 in Pubmed and 2 in Web of Science). In the Scopus AI search, the articles retrieved were split into 3 categories: references, foundational and related. Between the references and foundational categories, a duplicate was identified. In the traditional search, the Pubmed article was a duplicate of one of the Web of science articles. In the Scopus AI search, 1 book chapter and 4 reviews were retrieved. The remaining Web of Science article was also a review. When matching between the 2 batches (Scopus AI and Traditional search), no duplicate was identified. While the analysis and review of the articles was performed automatically by the Scopus AI tool, for a human to assess the articles, 5 more needed to be excluded as 2 were in German, 1 in Chinese, 1 in Croatian and 1 in Italian. Hence, the analysis included 17 articles.

**Figure 2:**
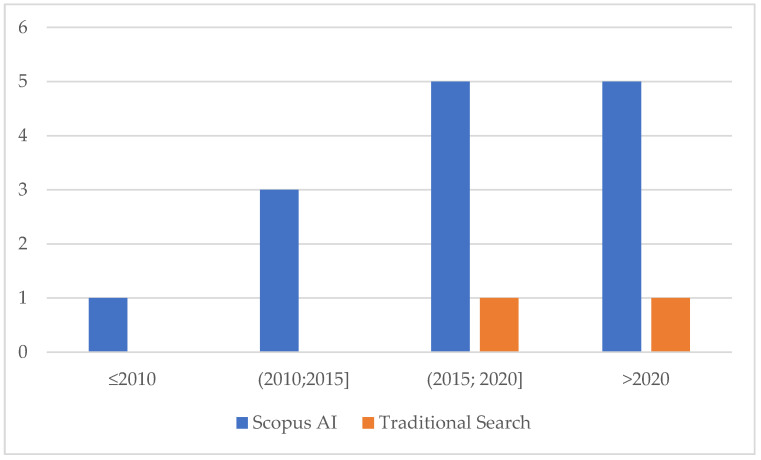
Distribution of publication years.

Based on the year of publication, most of the publications happened in the last decade (Figure 2). The time to complete the search was similar, at 30 min each, and the data was extracted into an excel spreadsheet.

Assessment of the results was performed automatically by Scopus AI, which provided the structured methodology presented below; meanwhile, for the 2 articles in the traditional search, the assessment had to be performed manually.

**Figure 3:**
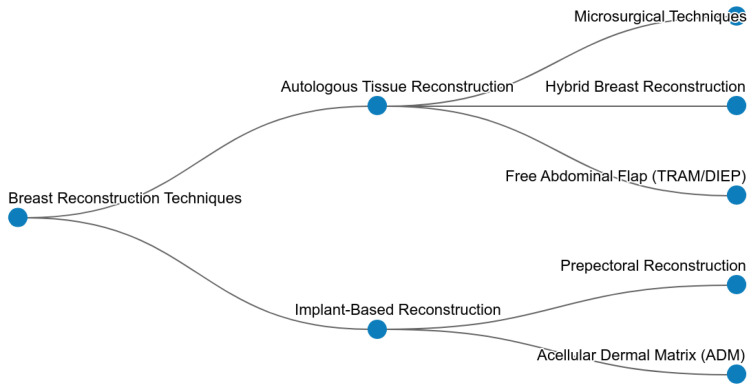
Scopus AI Concept Map.

In terms of results, Scopus AI provided not only a list of articles but also a summary (Table 1 and Table 2), an expanded summary (Table 3), a concept map (Figure 3), a list of emerging themes in beta version (Table 4) and the query of going deeper into the understanding of the subject in 3 different areas (Table 5).

In conclusion, primary prepectoral breast reconstruction after radical mastectomy offers several benefits, including reduced postoperative pain, improved esthetic outcomes, and high patient satisfaction. However, careful patient selection and management of potential complications are essential for optimal results.

Based on the query, I will provide a summary that covers the surgical techniques, advantages and disadvantages, patient selection considerations, and long-term outcomes and complications associated with primary prepectoral breast reconstruction after radical mastectomy.

In conclusion, primary prepectoral breast reconstruction after radical mastectomy involves various surgical techniques, each with its own set of advantages and disadvantages. By placing the implant above the pectoral muscle, this method reduces postoperative pain, shortens recovery time, and eliminates animation deformity, improving both functional and esthetic outcomes. Unlike submuscular placement, it avoids disruption of muscle function and allows for a more natural contour, particularly in direct-to-implant procedures. Patient selection considerations and long-term outcomes and complications are important factors to consider when opting for this reconstruction method. However, it is important to note that the evidence is limited, and further research is needed to fully understand the implications of prepectoral breast reconstruction.

## 4. Discussion

The results of our study demonstrate a significant disparity in the number of articles retrieved by Scopus AI and traditional keyword searches. Scopus AI, with its advanced natural language processing capabilities, identified 19 relevant articles, while the traditional approach yielded only 3. This substantial difference underscores the efficiency and potential of AI-powered tools in expanding the scope of literature reviews. However, it is crucial to acknowledge the potential drawbacks of relying solely on AI-generated results. The sheer volume of articles retrieved by Scopus AI can be overwhelming, particularly for researchers with limited expertise in the field. This abundance of information may lead to information overload, making it challenging to identify the most relevant and credible studies. Furthermore, the accuracy and relevance of AI-generated results can be influenced by the quality of the underlying algorithms and the specificity of the search queries. Strengths and Limitations: Discuss the strengths and limitations of using Scopus AI versus traditional PubMed keyword searches for conducting literature reviews.

Scopus AI retrieved several non-English and irrelevant records, a finding that likely reflects the platform’s algorithmic emphasis on maximizing recall rather than specificity. While this broad retrieval strategy increases the likelihood of capturing a more diverse body of literature, it also generates additional noise, requiring human screening to ensure that only relevant studies are retained. This characteristic underscores both the strengths and limitations of AI-assisted searching: although capable of widening the search net beyond conventional approaches, it still depends on critical human oversight to filter results. Future refinements in search prompt design, together with ongoing algorithmic development, may improve specificity, reduce extraneous outputs, and ultimately enhance the reliability and efficiency of AI-based literature searches.

The clinical relevance of prepectoral breast reconstruction makes it a suitable test case for evaluating search strategies; nonetheless, the primary aim of this study is to assess the performance of Scopus AI in comparison with traditional methods [3,18].

Artificial intelligence offers promising applications in this domain. In preoperative planning for DIEP flap reconstruction—a technique using abdominal perforators—AI-assisted CTA analysis has reduced planning time by approximately two hours per patient, with comparable accuracy to expert radiologists [19]. Furthermore, machine learning models predicting flap failure risk demonstrated moderate success (AUC~0.77), potentially aiding surgeons in personalized risk stratification [20].

Beyond planning, AI is making inroads into postoperative monitoring and patient care. Systems integrating wearable sensors and computer vision algorithms can detect early signs of complications—such as wound infection or compromised perfusion—potentially alerting clinicians before clinical symptoms appear [21]. Additionally, AI has been used to optimize pain management, analyze patient-reported outcomes, and support decision-making dashboards for surgeons [21].

Despite these advances, several challenges must be addressed before AI can be widely implemented in breast reconstruction. These include the need for large validated datasets, standardized protocols, transparency in algorithms, and thorough evaluation of clinical utility and cost-effectiveness. Most importantly, AI should complement—not replace—the expert judgment of plastic surgeons, who integrate nuanced clinical and patient-specific factors into surgical planning and care.

### 4.1. Implications for Future Research

The comparison between Scopus AI and traditional keyword search methodologies reveals significant insights that can be applied to future research topics, particularly in the field of breast reconstruction.

### 4.2. Enhanced Comprehensiveness

Scopus AI’s ability to retrieve a higher number of articles (19) compared to traditional keyword searches (3) demonstrates its potential to provide a more comprehensive overview of the literature. This can be particularly beneficial for emerging research topics where a broad understanding of existing studies is crucial. Researchers can leverage Scopus AI to ensure they are not missing out on relevant studies, thereby enhancing the depth and breadth of their literature reviews.

### 4.3. Improved Accessibility for Novices

For researchers with limited knowledge and understanding of a topic, Scopus AI can serve as a valuable tool to access a wide array of perspectives and findings. This can help build a solid foundation of knowledge and reduce the risk of bias that might arise from a more limited search. However, it is essential to provide guidance on how to navigate and filter the large volume of information to avoid potential overwhelm and confusion.

### 4.4. Focused and Manageable Results

While Scopus AI offers a comprehensive search, traditional keyword searches can provide more focused and manageable results. This can be advantageous for researchers who prefer a more targeted approach or those who are already familiar with the topic and seek specific studies. Combining both methodologies can offer a balanced approach, where Scopus AI is used for initial broad searches, followed by traditional keyword searches for more refined results.

### 4.5. Impact on Breast Reconstruction Research

In the field of breast reconstruction, the application of these methodologies can significantly impact the quality and scope of research. Scopus AI can help identify a wide range of studies, including those from diverse geographical regions and varying methodologies, contributing to a more holistic understanding of the field. This can lead to the identification of new trends, gaps in the literature, and potential areas for future research.

### 4.6. Encouraging Interdisciplinary Research

The comprehensive nature of Scopus AI can also encourage interdisciplinary research by uncovering studies from related fields that might not be immediately apparent through traditional keyword searches. This can foster collaboration between different disciplines, leading to innovative approaches and solutions in breast reconstruction.

### 4.7. Limitations

This study has several limitations that should be acknowledged. First, the comparison was limited to Scopus AI and a single PubMed keyword-based search conducted by the authors. This approach does not capture the full breadth of traditional search strategies; a more robust design would involve multiple databases, diverse query formulations, and independent reviewers performing parallel searches. Second, the absence of overlap between Scopus AI and PubMed results underscores the incomplete coverage of each method individually, highlighting the value of combining different approaches to achieve a more comprehensive literature review. Third, although Scopus AI retrieved a greater number of records, this apparent advantage must be interpreted cautiously, as it may reflect differences in query construction and database indexing rather than an inherent superiority of the tool. Fourth, the analysis represents a single time point (28 May 2025), and thus does not account for temporal variations in search results arising from ongoing updates in indexing or algorithmic changes in AI tools. Fifth, the study focused exclusively on one clinical topic—primary prepectoral breast reconstruction after radical mastectomy—limiting the generalizability of the findings to other surgical fields or AI platforms.

The reliance on a single AI tool (Scopus AI) may have introduced indexing bias, particularly favoring journals within the Elsevier portfolio. Furthermore, the exclusion of other major bibliographic databases (e.g., Ovid, Google Scholar) as well as gray literature represents an additional limitation. This restriction narrows the scope of the search and may limit the generalizability of the findings. Future research should address these issues by incorporating multiple AI platforms, expanding database coverage, and including gray literature sources. Such efforts would reduce potential bias and provide a more balanced and comprehensive evaluation of AI-assisted search strategies.

Finally, while two authors independently screened and selected eligible studies, the process inevitably involved human judgment, introducing potential subjectivity and bias despite efforts to resolve disagreements by consensus. Overall, these limitations remind us that this work should be seen as a first step rather than a final verdict on AI-assisted searching. Our goal was to test feasibility, not to deliver a definitive assessment of such tools, and the results should therefore be read with appropriate caution. To move beyond this exploratory stage, larger studies that draw on multiple AI platforms, cover a wider range of databases, and address different clinical areas will be needed. Only through such efforts will it be possible to confirm—or challenge—our initial observations and build a more reliable evidence base for the role of AI in literature searches.

## 5. Conclusions

### 5.1. Efficiency and Comprehensiveness of Scopus AI

Scopus AI demonstrated a significant advantage in retrieving a higher number of relevant articles (19) compared to a traditional keyword search (3). This highlights the efficiency and comprehensiveness of AI-powered tools in conducting literature reviews, especially for emerging research topics where a broad understanding of existing studies is crucial.

### 5.2. Impact on Researchers with Limited Expertise

For researchers with limited knowledge and understanding of the topic, Scopus AI can provide a valuable tool to access a wide array of perspectives and findings. This can help build a solid foundation of knowledge and reduce the risk of bias that might arise from a more limited search. However, the large volume of information retrieved by Scopus AI can be overwhelming, potentially leading to information overload and confusion.

### 5.3. Quality and Relevance of Results

The quality and relevance of the results obtained from Scopus AI were generally high, with the tool providing structured summaries, concept maps, and emerging themes. This automated analysis can save researchers significant time and effort. However, the accuracy and relevance of AI-generated results can be influenced by the quality of the underlying algorithms and the specificity of the search queries.

### 5.4. Manual Assessment in Traditional Searches

Traditional keyword searches, while yielding fewer articles, provided more focused and manageable results. The manual assessment of these articles ensured that only the most relevant studies were included in the analysis. This approach can be advantageous for researchers who prefer a more targeted approach or those who are already familiar with the topic and seek specific studies.

### 5.5. Future Research

The integration of Scopus AI and traditional keyword search methodologies offers a powerful combination for future research. By leveraging the strengths of both approaches, researchers can enhance the comprehensiveness, accessibility, and focus of their literature reviews. This can ultimately lead to more robust and impactful research in the field of breast reconstruction and beyond.

### 5.6. Interdisciplinary Research

The comprehensive nature of Scopus AI can encourage interdisciplinary research by uncovering studies from related fields that might not be immediately apparent through traditional keyword searches. This can foster collaboration between different disciplines, leading to innovative approaches and solutions in breast reconstruction.

The study underscores the potential of AI-powered tools like Scopus AI to revolutionize literature reviews by providing a more comprehensive and efficient search process. However, it also highlights the importance of balancing AI-generated results with traditional methods to ensure the relevance and manageability of the information, particularly for researchers with limited expertise. The combined use of both methodologies can significantly enhance the quality and scope of research in breast reconstruction and other fields.

### 5.7. General and Contextual Considerations

AI-driven platforms such as Scopus AI hold considerable promise for accelerating and broadening literature reviews, providing structured outputs that can reduce researchers’ workload. Nevertheless, our findings indicate that AI-based searches cannot yet replace traditional methods. Rather, they should be viewed as complementary tools that enhance—but do not substitute—the rigor of manual, keyword-based approaches. Future studies should build on this work by integrating broader databases, employing diverse search strategies, involving independent reviewers, and repeating searches at different time points to capture updates in indexing and AI outputs. These efforts will be essential to establish a balanced, evidence-based framework for incorporating AI tools into scholarly practice while safeguarding the accuracy, reliability, and transparency central to scientific inquiry.

## Figures and Tables

**Table 1:** Scopus AI Summary.

Category	Explanation
Primary Prepectoral Breast Reconstruction After Radical Mastectomy	Prepectoral breast reconstruction (PPBR) has gained popularity as a technique for breast reconstruction following mastectomy due to its numerous advantages over traditional subpectoral methods. This summary provides an overview of the benefits, complications, and outcomes associated with PPBR based on recent studies.
Advantages of Prepectoral Breast Reconstruction:	Reduced Postoperative Pain: PPBR avoids the dissection of the pectoralis major muscle, which significantly reduces postoperative pain and the risk of animation deformity compared to subpectoral reconstruction [1,2,3]. Improved Esthetic Outcomes: The use of acellular dermal matrices (ADMs) and advancements in implant technology have enhanced the cosmetic results of PPBR, making it a preferred option for many patients [4,5]. Lower Complication Rates: Studies have shown that PPBR is associated with lower rates of capsular contracture and implant migration, especially in patients undergoing postmastectomy radiation therapy (PMRT) [6,7].
Complications and Considerations:	Infection and Seroma: While PPBR is generally safe, it is not without complications. The most common issues include surgical site infections and seromas, although these are manageable and occur at rates comparable to subpectoral techniques [2,6,8]. Patient Selection: Proper patient selection is crucial for the success of PPBR. Factors such as body mass index (BMI), comorbidities, and the need for adjuvant therapies like radiation must be considered [2,9].
Clinical Outcomes:	Patient Satisfaction: Studies indicate high levels of patient satisfaction with PPBR, with many patients reporting excellent esthetic outcomes and improved quality of life [4,10,11]. Functional Recovery: PPBR has been shown to facilitate quicker functional recovery, with patients experiencing less pain and disability in the early postoperative period compared to those undergoing subpectoral reconstruction [3]. Long-term Safety: Preliminary results suggest that PPBR is a safe and effective method for breast reconstruction, with minimal long-term complications and good integration of ADMs even in irradiated tissues [7,12].

**Table 2:** Scopus AI Summary on Cmparison of Prepectoral and Subpectoral Techniques.

Aspect	Prepectoral	Subpectoral
Postoperative Pain	Lower [1,2,3]	Higher [1,2,3]
Esthetic Outcomes	Improved [4,5]	Comparable [4,5]
Complication Rates	Lower capsular contracture [6,7]	Similar overall [2,6,8]
Patient Satisfaction	High [4,10,11]	High [4,10,11]
Functional Recovery	Faster [3]	Slower [3]

**Table 3:** Scopus AI Expanded Summary.

Category	Explanation
Surgical Techniques:	Primary Reconstruction Techniques: Primary breast reconstruction can be performed using various techniques, including implant reconstructions, latissimus dorsi flap (LDF) with or without implants, and transversal rectus abdominis myocutaneous (TRAM) flap, with implant reconstructions being the most common [13,14,15]. Prepectoral Reconstruction: Prepectoral breast reconstruction involves placing the implant above the pectoralis major muscle, offering advantages such as reduced animation deformity, pain, and weakness compared to submuscular implants [6,16].
Advantages and Disadvantages:	PAdvantages of Prepectoral Reconstruction: Prepectoral reconstruction has been associated with better esthetic outcomes, reduced pain, and elimination of animation deformity compared to submuscular reconstruction [6,16]. Disadvantages of Prepectoral Reconstruction: However, prepectoral reconstructions have been linked to higher rates of surgical-site occurrences, capsular contracture, and explantation compared to submuscular reconstructions [16].
Patient Selection Considerations:	Considerations for Reconstruction Approach: The choice of reconstruction approach depends on factors such as breast volume, patient’s wish, and experience of the surgical team [13,14]. Timing of Reconstruction: Immediate breast reconstruction is preferable for patients with a low risk of requiring postmastectomy radiation therapy, while delayed reconstruction may be preferable for patients deemed to require postmastectomy radiation therapy to avoid difficulties associated with radiation delivery after immediate reconstruction [17].
Long-term Outcomes and Complications:	Complications: Complications associated with prepectoral breast reconstruction include seroma, hematoma, infections, partial flap necrosis, implant rejection, hypertrophic scarring, and hernias at the flap elevation site [13,14]. Outcomes: Despite the complications, prepectoral breast reconstruction with acellular dermal matrices in the setting of postmastectomy radiation therapy has been reported to be a safe and successful surgical option resulting in excellent clinical outcomes [6].

**Table 4:** Scopus AI Expanded Summary detailed.

Theme Name (with References)	Definition	Potential Hypothesis (with Hyperlinks)
Patient Satisfaction and Quality of Life in Prepectoral Breast Reconstruction	The consistent interest in patient satisfaction and quality of life following prepectoral breast reconstruction highlights the importance of understanding patient-centered outcomes. This theme is crucial for improving surgical techniques and postoperative care to enhance patient experiences and satisfaction.	Prepectoral breast reconstruction results in higher patient satisfaction compared to subpectoral reconstruction Quality of life metrics improve significantly within the first year post-prepectoral breast reconstruction
Advancements in Acellular Dermal Matrix (ADM) Utilization	The ongoing research into the use of acellular dermal matrices (ADM) in prepectoral breast reconstruction indicates a sustained interest in optimizing surgical outcomes and reducing complications. This theme is vital for developing new materials and techniques to improve the safety and efficacy of breast reconstruction procedures.	The use of ADM in prepectoral breast reconstruction reduces the incidence of postoperative complications Different types of ADM materials have varying impacts on the long-term outcomes of prepectoral breast reconstruction
Innovations in Imaging and Measurement Techniques for Breast Reconstruction	The rising interest in advanced imaging and measurement techniques for prepectoral breast reconstruction reflects the need for precise preoperative planning and postoperative assessment. These innovations are crucial for improving surgical accuracy and patient outcomes.	3D imaging techniques significantly enhance the accuracy of preoperative planning in prepectoral breast reconstruction Advanced imaging modalities improve the assessment of postoperative outcomes in breast reconstruction patients
Nipple-Sparing Mastectomy and Prepectoral Reconstruction	The increasing focus on nipple-sparing mastectomy combined with prepectoral reconstruction highlights the trend towards more conservative and esthetically favorable surgical options. This approach aims to preserve the nipple-areolar complex while providing effective reconstruction, which is significant for patient satisfaction and cosmetic outcomes.	Nipple-sparing mastectomy with prepectoral reconstruction results in better esthetic outcomes compared to traditional methods Patients undergoing nipple-sparing mastectomy with prepectoral reconstruction report higher satisfaction rates
Prepectoral Tissue Expander Techniques	The novel focus on prepectoral tissue expander techniques without the use of acellular dermal matrix (ADM) represents a new direction in breast reconstruction research. This theme explores the feasibility, safety, and outcomes of using tissue expanders in a prepectoral plane, which could offer alternative options for patients and surgeons.	Prepectoral tissue expander placement without ADM is as safe and effective as traditional methods Patients with prepectoral tissue expanders experience fewer complications compared to those with subpectoral expanders

**Table 5:** Go Deeper.

What are the key considerations for patient selection in primary prepectoral breast reconstruction? How does primary prepectoral breast reconstruction compare to subpectoral reconstruction in terms of outcomes and complications? What are the potential challenges and limitations of primary prepectoral breast reconstruction after radical mastectomy?

## Data Availability

No new data were created or analyzed in this study.
